# Sustentacular Cell Enwrapment of Olfactory Receptor Neuronal Dendrites: An Update

**DOI:** 10.3390/genes11050493

**Published:** 2020-04-30

**Authors:** Fengyi Liang

**Affiliations:** Department of Anatomy, Yong Loo Lin School of Medicine, National University of Singapore, 4 Medical Drive, Singapore 117594, Singapore; antlfy@nus.edu.sg; Tel.: +65-6516-1936

**Keywords:** olfactory receptor neuron (ORN), dendrite, enwrapment, olfactory sustentacular cell (OSC)

## Abstract

The pseudostratified olfactory epithelium (OE) may histologically appear relatively simple, but the cytological relations among its cell types, especially those between olfactory receptor neurons (ORNs) and olfactory sustentacular cells (OSCs), prove more complex and variable than previously believed. Adding to the complexity is the short lifespan, persistent neurogenesis, and continuous rewiring of the ORNs. Contrary to the common belief that ORN dendrites are mostly positioned between OSCs, recent findings indicate a sustentacular cell enwrapped configuration for a majority of mature ORN dendrites at the superficial layer of the OE. After vertically sprouting out from the borderlines between OSCs, most of the immature ORN dendrites undergo a process of sideways migration and terminal maturation to become completely invaginated into and enwrapped by OSCs. Trailing the course of the dendritic sideways migration is the mesodendrite (mesentery of the enwrapped dendrite) made of closely apposed, cell junction connected plasma membrane layers of neighboring folds of the host sustentacular cell. Only a minority of the mature ORN dendrites at the OE apical surface are found at the borderlines between OSCs (unwrapped). Below I give a brief update on the cytoarchitectonic relations between the ORNs and OSCs of the OE. Emphasis is placed on the enwrapment of ORN dendrites by OSCs, on the sideways migration of immature ORN dendrites after emerging from the OE surface, and on the terminal maturation of the ORNs. Functional implications of ORN dendrite enwrapment and a comparison with myelination or Remak’s bundling of axons or axodendrites in the central and peripheral nervous system are also discussed.

## 1. Introduction

The main olfactory epithelium (OE) in mammals consists of a relatively simple pseudostratified epithelial cell layer lining the superior/dorsal part of the nasal cavity. Its basic cytological features have been known since at least the middle of the 19th century [1,2]. Apart from the bipolar olfactory receptor neurons (ORNs), the OE also comprises olfactory sustentacular cells (OSCs), horizontal and globose basal cells, ductal cells of Bowman’s glands, and sporadic microvillar cells [3,4,5,6,7,8,9,10,11]. 

The ORNs are undoubtedly the OE’s parenchymal cells responsible for olfactory reception and transduction. These bipolar neuronal cells are directly exposed at the dendritic end to the nasal mucus and potentially harmful agents or microorganisms in the ambient air of the nasal cavity. At the axonal pole, the ORNs are synaptically connected to the olfactory bulb of the central nervous system (CNS) [6,8]. According to their expression of G-protein-coupled odorant receptors, ORNs in the OE could be differentiated into hundreds of subsets, with each subset usually showing only one phenotype of odorant receptor proteins; axons from the same ORN subset converge to selectively project to the same one or a few glomeruli of the olfactory bulb [12,13]. The OSCs are believed to be partly epithelial and partly glial, functioning as major physical, metabolic, secretory, absorptive, phagocytic, and diverse other supports for the ORNs and the OE overall [3,4,10,11,14,15,16,17]. The globose and horizontal basal cells represent essentially the precursor or stem cells of the OE that could give rise to other OE cell types, especially ORNs that have a relatively short lifespan of only a few weeks, and therefore are continuously replaced throughout life of the organism [5,18,19,20,21]. The nature and roles of the OE microvillar cells remain unclear [7,8], but recent findings point toward modulatory and maintenance functions for these cells in olfactory reception, ORN apoptosis and regeneration, or in OE aging [22,23,24,25].

## 2. Early Cytological and Cytoarchitectonic Studies of the Olfactory Epithelium

**Figure 1:**
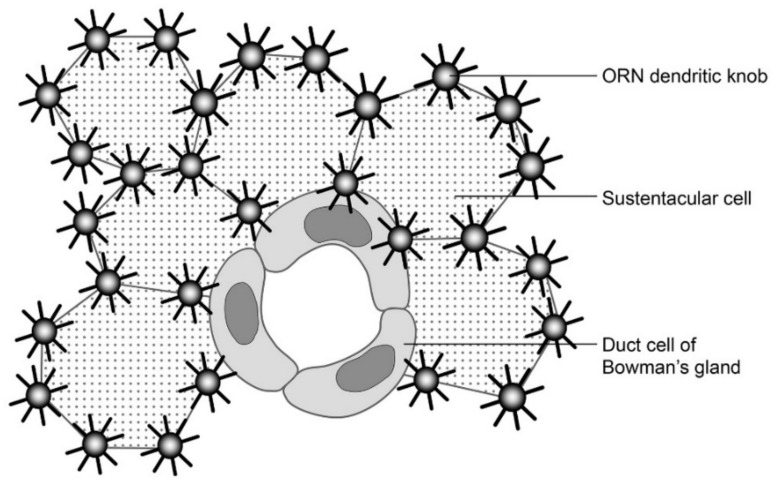
A schematic diagram illustrating major cell types and their relations on the tangential view of the luminal surface of olfactory epithelium of the rabbit. Re-drawn with permits from [2].

The OE serves both epithelial and special sense (olfactory) functions. As an epithelium, it protects and separates deeper structures from the air of the nasal cavity. As a special sense organ, the OE is the site of olfactory reception and signal transduction, and ORN axonal projection to the olfactory bulb transmits olfaction signals to the CNS. These dual roles of the OE are structurally subserved not only by the OE cell types, but also by the cytoarchitectonic organization and interrelations among the cells, especially those between the ORNs and OSCs. Indeed, the study of OE cytology and cytoarchitecture has a long history in various vertebrate species ranging from fish to man. Earlier literature concerning this topic has been extensively reviewed [2,26]. Before the 1970s, it was generally assumed that mammalian ORN dendrites were located at the borderlines between OSCs [2,26], as typically illustrated in Figure 1. 

Breipohl et al. [3] first reported “a few” sustentacular cell-enclosed ORN dendrites, apart from the “normal” majority of ORN dendrites between OSCs in the OE of the mouse and goldfish. The enclosed ORN dendrites in the goldfish occasionally showed spiral-shaped casing around. In the mouse OE, several ORN dendrites appeared enclosed in one sustentacular cell apical process. The authors further suggested similarities among sustentacular cell enclosure of ORN dendrites, myelination of neuronal axons by Schwann cells or oligodendroglia, and surrounding of optical receptor cell processes by retinal pigment epithelial cells [3].

These findings by Breipohl et al. [3] were subsequently confirmed in the human and rat OE [9,27,28]. By using scanning and transmission electron microscopy, Morrison and Costanzo [27] stated that human ORN cell bodies and dendrites were partially surrounded by OSCs, and ORN axons were surrounded by cellular extensions or sleeve-like processes of OSCs at the basal layer of the OE. Similarly, the scanning electron microscopic study of the rat OE by Nomura and coworkers [28] described groups of ORN cell bodies aligned along vertical columns and roughly incompletely wrapped by flat processes of OSCs. Mature ORN dendritic shafts were often loosely incompletely invested by plicate processes or longitudinal folds of OSCs, and immature ORNs at the basal layer of the OE were also found to be partly enclosed by foot processes of OSCs. Dendrites of immature ORNs, however, were usually independent of sustentacular cell investment. The investment of ORN dendrites by OSCs was suggested to serve a function for micro-electrical isolation, but not so much for guiding initial growth of immature ORN dendrites [28]. More recently, in the study of cell junctions in the OE and olfactory fila, Steinke and colleagues [9] also clearly demonstrated the existence of ORN dendrites embedded into OSCs, in addition to dendrites situated between two or several adjacent supporting cells. 

**Figure 2:**
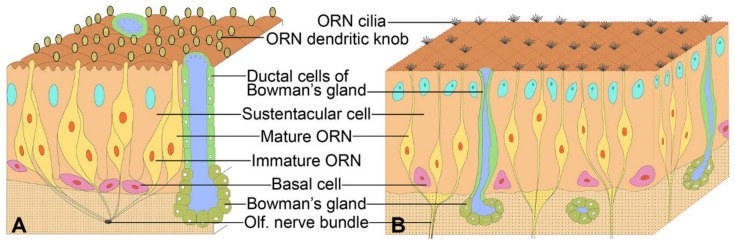
Schematic illustrations of the olfactory epithelium cytology as described in some of the recent publications [29,30]. Both illustrations clearly assumed olfactory receptor neuron (ORN) dendrite positions between olfactory sustentacular cells (OSCs). (**A**) Re-drawn and modified with permits from [29] and (**B**) re-drawn from [30] with permits.

In spite of these and other works, up to the present day it is still generally believed that an overwhelming majority of ORN dendrites in the OE are located between OSCs [29,30] (Figure 2), and the wrapping of ORN dendrites by OSCs, if recognized at all, has been characterized as occasional and partial [31,32,33,34].

In the following section, I would focus on some of the recent findings on the cytoarchitectonic relations of ORN dendrites and OSCs of adult rat OE. Although the relations in many other vertebrates including man remain largely unclear, we have good reasons to believe that these findings in the rat most likely apply, at least partially, to the OE of many other species, given the aforementioned previous findings, and the fact that the basic cytology and cytoarchitectonic organization of the OE are rather conserved all through the vertebrates [2,26]. 

## 3. A Majority of Olfactory Receptor Neuron Dendrites are Enwrapped by Sustentacular Cells

**Figure 3:**
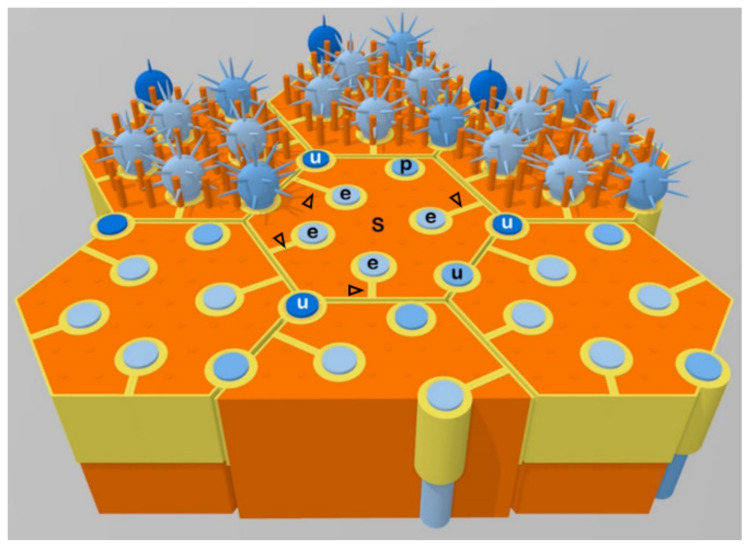
A three-dimensional diagram to show the rat olfactory epithelium (OE) apical layer. Six and a half OSCs (S, orange) and related ORN dendrites are shown, three with intact OSC microvilli, related dendritic knobs, and cilia (top row), and the rest having the OSC microvilli and related dendritic knobs removed to better illustrate relations among the cells. Newly emerged ORN dendrites are immature (highly immunoreactive for class-III β-tubulin) and located at the borderlines between OSC apices (unwrapped, three of them being labeled by “u” in white). Along the course of further maturation (as marked by progressively weakening class-III β-tubulin immunoreactivity), most of the dendrites undergo sideways migration and become enwrapped (“e”) by OSCs, but a few remain unwrapped upon maturity (one being labeled by ‘u’ in black). Partially-wrapped ORN dendrites (one labeled by “p”) may represent intermediate stages from unwrapped immature to enwrapped mature status. The apical junctional complex (yellow) can be distinguished into those homotypic cell–cell junctions between OSCs, heterotypic junctions between OSCs and ORN dendrites, and autotypic junctions that link neighboring pleats or folds of the same OSC, and form the mesenteries of enwrapped dendrites (mesodendrites, arrowheads). Modified with permits from [11].

Under a confocal or electron microscope, the cytoarchitectonic relations between cross-sectioned ORN dendrites and OSCs or other cells could be readily visualized on tangential or oblique histological sections of the OE superficial layer stained by ZO1 (zonula occludens-1, a tight junction protein) immunohistochemistry or actin cytoskeleton histochemistry. Apart from unwrapped ORN dendrites (examples being labeled by “u” in Figure 3) located at the borderlines between OSCs or occasionally between an OSC and a microvillar cell, there exist ORN dendrites completely-wrapped (enwrapped) or partially-wrapped by OSCs. A cross-sectioned enwrapped dendrite (examples being labeled by “e” in Figure 3) appears circular, completely invaginated into a vertical passage barrel inside a single OSC, and linked to the borderline between the host and a neighboring OSC by a mesentery of the enwrapped dendrite (mesodendrite) (Figure 3, arrowheads). A partially-wrapped dendrite (one example being labeled by “p” in Figure 3) is largely invaginated into a deep vertical groove on the side of an OSC, but a small part (less than 1/4) of its surface remains directly linked to the inter-sustentacular borderline. Therefore, partially-wrapped dendrites display no observable mesodendrites. 

In summary, based on their cytoarchitectonic relations with OSCs, ORN dendrites at the OE superficial layer could be subtyped as follows: i) The unwrapped dendrites are positioned at the inter-sustentacular cell borderlines, ii) The enwrapped dendrites are each enclosed in a vertical passage barrel within a single OSC. The plasma membrane of the enwrapped dendrite is closely apposed to, and linked by, intercellular junctions with host OSC plasma membrane at the dendritic passage barrel. The mesentery of an enwrapped dendrite (mesodendrite) tethers the dendritic passage barrel to the side of the host OSC, and is formed by closely apposed, cell junction-connected plasma membrane layers of neighboring pleats/thick folds of the host OSC. Up to six enwrapped dendrites could be seen in the confine of a single OSC apical process. A possible mechanism for the formation of the mesodendrite will be discussed below, iii) A partially wrapped dendrite is almost, but not yet completely, wrapped by an OSC. As discussed below, the partially wrapped dendrites probably represent the intermediate stage of ORN dendrites progressing from unwrapped to enwrapped status.

The above-observed cytoarchitectonic relations between ORN dendrites and OSCs confirm previous findings in the mouse, goldfish, rat, or man [3,9,27,28]. It then came, quite unexpectedly, to discover that a majority (~54%) of the ORN dendrites at the rat OE superficial layer are actually enwrapped by OSCs. Unwrapped ORN dendrites account for only ~28%, and partially-wrapped dendrites were even fewer (~18%) [11]. These quantitative data seemingly contradict the conventional belief of a “normal” unwrapped positioning for an absolute majority of ORN dendrites. The discrepancy might be attributed to the fact that few quantitative analyses were attempted previously. The earlier notion seemed mainly based on qualitative observations by using high-power electron microscopy [3,9,27,28]. 

Cell junctions in the OE have been extensively studied, by using transmission or scanning electron microscopy, confocal microscopy, immunohistochemistry, molecular biology, and other approaches. The OE possesses various junctional structures between cells or cell parts of a same cell. Typical apical junctional belt of zonula occludens tight junctions and zonula adherens junctions is present at the OE apical layer [3,9,11,35,36]. There are puncta adherentia and desmosomes between the basolateral sides of OE cells [9,37]. Of particular interest, the adherens junctions between the ORNs and OSCs appear different from those between OSCs or between sustentacular and microvillar cells [9]. Gap junctions have also been reported in the OE, but molecular details and cellular distribution of gap junctions in the ORNs and OSCs remain unclear [38,39]. 

## 4. The Enwrapment, Sideways Migration and Terminal Maturation of ORN Dendrites

Class-III β-tubulin (Tuj1 immunoreactivity) is a known marker of immature ORNs. Its expression largely stops in ORN cell bodies and greatly weakens in ORN dendrites when ORNs start to show positivity for olfactory marker protein (a marker of mature ORNs) [9,40]. Thus, weak Tuj1 immunoreactivity indicates maturity of ORNs. In accordance with this notion, it was observed in the rat OE that intensely Tuj1-immunoreactive ORN cell bodies were mostly located near the basement membrane, whereas very weakly Tuj1-positive ORN cell bodies were mostly located in the OE middle layer [11]. 

ORN dendrites at the OE superficial layer also display significantly variable Tuj1 immunoreactivity intensities. Surprisingly, correlation of Tuj1 immunoreactivity intensities with wrapping status of ORN dendrites revealed that essentially all enwrapped dendrites have weak Tuj1 immunoreactivity. Strongly Tuj1-immunoreactive ORN dendrites are mostly located at the inter-sustentacular cell borderlines, and thus belong to the unwrapped subtype. A small number of the partially wrapped dendrites also exhibit high Tuj1 immunoreactivity. In view of the abovementioned reports of high Tuj1 immunoreactivity marking immature ORNs and weak Tuj1 immunoreactivity marking olfactory marker protein-expressing ORNs, these results indicate that the sustentacular cell-enwrapped dendrites mostly, if not all, belong to mature ORNs. The unwrapped and partially wrapped subtypes include practically all of the highly Tuj1-immunoreactive immature ORN dendrites and a portion of the low Tuj1-immunoreactive mature ORN dendrites. Quantitatively, the highly Tuj1-immunoreactive immature dendrites account for ~13% (10% unwrapped and 3% partially-wrapped) of all ORN dendrites at the OE apical surface, and the low-Tuj1-immunoreactive mature dendrites make up ~87% (54% enwrapped, 18% unwrapped, and 15% partially-wrapped). Overall, it appears that immature dendrites of newly generated ORNs mostly or all emerge from the borderlines between OSCs (unwrapped). This notion is consistent with previous observations by Nomura and coworkers [28] stating that “the dendrite of mature neurons was often wrapped by the supporting cells, while that of immature neurons was usually rather independent from the supporting cells”. 

The question then arises as to how the unwrapped immature ORN dendrites further mature after arriving at the OE apical surface, and eventually become mostly enwrapped [11]. Based on the presence of a mesentery (mesodendrite) trailing each enwrapped dendrite, the enwrapment appears to involve a sideways migration process of the newly-emerged immature dendrites from the inter-sustentacular borderlines to the intra-sustentacular enwrapped positions. The diversion point of the mesodendrite from the inter-sustentacular borderline was most likely the starting point, whereas the trajectory of the mesodendrite probably represents the path of the sideways migration process [11]. All enwrapped dendrites seem derived from the unwrapped. In other words, probably no ORN dendrite is enwrapped and mature upon first arrival at the OE luminal surface. This notion is supported by previous data indicating a loss of strong Tuj1 immunoreactivity in ORN dendrites only after the dendrites have inserted into the OE surface and started to produce olfactory marker protein [9]. 

Thus, the sideways migration appears a terminal maturation process undertaken by all enwrapped ORN dendrites. From this point of view, some or most of the partially wrapped dendrites possibly represent those at the intermediate stage of sideways migration from immature unwrapped to mature enwrapped status. Judging from the proportions of mature and immature ORN dendrites in the partially wrapped group (15% and 3%, respectively), it seems that most of the dendrites destined for OSC enwrapment could complete the terminal maturation process before reaching the final enwrapped position. 

It should be noted that some 18% ORN dendrites remain unwrapped after maturity, as judged by their low levels of Tuj1 immunoreactivity. Currently, it is unknown whether these dendrites have also to undergo the process of sideways migration (understandably along the direction of inter-sustentacular borderlines, if at all) for terminal maturation at the OE luminal surface, or these dendrites mature in situ at the sites of emerging to the OE surface. In any case, the presence of a fraction of mature ORN dendrites at the inter-sustentacular borderlines suggests that OSC enwrapment is not really indispensable for terminal maturation and essential functionality of ORN dendrites. It awaits future investigations to elucidate the exact functional roles of ORN dendritic enwrapment. Among other possibilities, the enwrapment could help evenly disperse and insulate ORN dendrites and dendritic knobs on the OE surface to enhance olfactory reception or discrimination, or organize the mature dendrites into subgroups and modular units of olfactory reception, transduction, sensitivity, receptor potential transmission or other attributes. OSCs, for example, are reactive to purinergic and cholinergic neurochemicals, produce endocannabinoids, generate long-lasting positive potentials or propagatable Ca^2+^ signals when stimulated, and therefore could potentially modulate activities of ORN dendrites within its confine [10,14,16,41,42,43,44]. 

Thus ORN maturation perceivably involves not only the conventionally known vertical migration-and-sprouting phase for the cell bodies and dendrites, but also the newly discerned sideways migration terminal maturation phase for the enwrapped dendrites at least. The precision and complexity of the former phase have been fully appreciated [12,13,45,46,47], whereas the latter phase still awaits more in-depth investigations and better understanding. Given an estimated 40-day average lifespan of mature ORNs [18,19,20,21], the average immature-to-mature turnover time for the sideways migration and terminal maturation of newly emerged immature ORN dendrites is estimated to be about 5.98 days, based on the 13:87 ratio for percentage of immature-to-mature ORN dendrites at the OE luminal surface. In other words, after arriving at the OE surface, immature ORN dendrites spend another 5–6 days to further mature and downregulate own Tuj1 immunoreactivity. In reality, the turnover time should be even shorter as the turnover rate from immature to mature dendrites is unlikely to be always 100%. 

## 5. Comparison of ORN Dendritic Enwrapment, Neurite Myelination, Remak’s Bundling, and Possible Molecular Mechanisms

As far as the enwrapment of ORN dendrites is concerned, the OSCs somehow share similarities with the Schwann cells and oligodendrocytes of the peripheral and central nervous systems, respectively. The enwrapped ORN dendrites are somehow reminiscent of the myelinated nerve fibers, whereas the unwrapped ORN dendrites are reminiscent of the unmyelinated fibers. This poses the question as to whether the olfaction reception, signal transduction, or receptor potential propagation of enwrapped mature ORN dendrites may differ from that of the unwrapped mature ORN dendrites. Action potential conductions, for example, greatly differ between myelinated and unmyelinated nerve fibers [48,49]. At present, it is equally unclear if there is systematic variation in some other structural and functional properties between enwrapped and unwrapped ORN dendrites.

Cytologically, the relation between the enwrapped ORN dendrites and host OSCs might more resemble that between the unmyelinated C fibers and Schwann cells in the Remak’s bundles of peripheral nerves, especially the sensory group-C fibers of the peripheral nervous system. If we consider direction of action or receptor potential propagation, the sensory C fibers in the peripheral nerves can indeed be viewed as dendrites or axodendrites of dorsal root ganglion neurons, much like the ORN dendrites. The so called mesaxon (mesentery of axon) associated with each of the C fibers in the Remak’s bundle also bears great resemblance to the mesenteries of enwrapped ORN dendrites (mesodendrites). Remak’s bundling of C fibers has possible functional roles in nerve development, differentiation or regeneration, and abnormalities of the Remak’s bundles have been implicated in neurological disorders like neuropathies [49,50]. It remains to be clarified if ORN dendrite enwrapment may have similar functional roles in the development, differentiation, or regeneration of ORNs, and if abnormalities of ORN dendrite enwrapment may result in olfaction dysfunctions such as anosmia, parosmia, or phantosmia. This is especially worth noting in relation to well-documented olfaction abnormalities in aging, neurodegenerative, or neuropsychiatric disorders like Alzheimer’s disease and schizophrenia [51,52,53,54,55,56,57,58].

At the molecular level, the ORNs share the common features of other neuronal cells of the nervous system, but the OSCs have been considered more as epithelial cells (as indicated by the cell type’s expression of such marker proteins as keratin 8, E-Cadherin and Keratin 18 [59]). Few molecular similarities with neuroglia (such as Schwann cells or oligodendroglia) have been reported of the OSCs, with the exception of the actin cytoskeleton-related ezrin-radixin-moesin (ERM) protein family that serve as well-known linker molecules between cellular plasma membrane and actin cytoskeleton. Both the actin cytoskeleton and the ERM proteins play important roles not only in various epithelial cells, but also in the Schwann cells and oligodendroglia (in an event of myelination and/or formation of the node of Ranvier [60,61,62,63,64]). The ERMs are also abundantly expressed in the OSCs, especially at the apices and microvilli of OSCs [65]. 

More interestingly, juxtanodin (ermin), a protein molecule that shares the C-terminal actin-binding motif of the ERMs but lacks the N-terminal FERM (4.1 protein-ERM) domain thereof, was originally reported a specific oligodendroglial myelinic protein and a regulator of oligodendroglial actin cytoskeleton dynamics [66,67,68,69]. Specific juxtanodin (ermin) expression has subsequently been revealed in the OSCs and in retinal pigment epithelial cells [17,70]. These data add to the molecular commonalties among the myelin-forming and special sense organ supporting cells, and strongly implicate the actin cytoskeleton and related proteins in regulating ORN dendrite wrapping by OSCs. These commonalities also suggest shared molecular mechanisms between myelination and enwrapment of ORN dendrites. The retinal photoreceptor neurons (rods and cones) notably are also partly surrounded by retinal pigment epithelial cells. However, rather than the sideways invagination of ORN dendrites into OSCs, the outer segments of retinal rods and cones approach the retinal pigment epithelial cells from the opposite direction, and interdigitate with the microvilli and protrusions of the latter [71].

## 6. Some Other Questions and Future Studies

The finding of ORN dendrite enwrapment and sideways migration updated our understanding of the OE cytoarchitecture. It also raised many new questions that should be addressed in future studies. First, in terms of phylogeny and ontogeny, quantitative data of ORN dendrite enwrapment and lateral migration maturation is not available for comparison among species, organisms, or developmental and aging stages of individual species. Qualitatively, it has been reported that the mouse has relatively more sustentacular cell-enclosed ORN dendrites than the goldfish, and sustentacular cell-enclosed ORN dendrites were seen not only in the adult mouse OE, but also in the fetus and newborn mice [3]. Concerning the latter, it should be noted that ORN dendrites in the fetus or newborn may not necessarily be immature, as olfactory marker protein-positive ORNs and dendrites are already present in the embryo [27]. Further investigations of the enwrapment and sideways migration of ORN dendrites in different species and developmental or aging stages would help understand the biology and functions of the OE.

Second, detailed cytoarchitectonic relations among the cell types at middle and basal layers of the OE, especially between OSC foot processes and ORN cell bodies and axons, remain unclear. Limited electron microscopic studies have suggested the presence of partially wrapped ORN cell bodies and axons [27,28]. Further investigation of the issue would elucidate if sustentacular wrapping/enclosure, complete or partial, has a role in development, differentiation or vertical migration guidance of immature ORNs, or in the elongation and selective bundling of ORN axons. 

More importantly, are there possible molecular or functional differences among the enwrapped, partially-wrapped and unwrapped mature ORN dendrites? Among others, possible differences in odorant receptor expression profiles remain elusive between the mature enwrapped and mature unwrapped ORN dendrites, among ORN dendrites wrapped within individual OSCs, or between ORN dendrites enwrapped by different OSCs. Moreover, are there variations in ORN dendrite enwrapment across different zones/regions of the OE in relation to the systematic variations of odorant receptor expression across the zones [12,13], or in relation to the different subsystems of odorant receptors like the class-I and class-II canonical odorant receptors or trace amine-associated receptors? [72,73,74,75,76] Further clarifications on these and related other issues would not only enlighten us on the biological and functional meaning of ORN dendrite enwrapment and differentiation, but may also implicate the wrapping status of ORN dendrites in organizing olfactory subsystems. 

Finally, what are the possible pathological changes of the ORN dendrite wrapping status and sideways migration in anosmia, parosmia, neurodegenerative, and neuropsychiatric disorders? Olfaction dysfunctions are frequent early manifestations of neuropsychiatric and aging-related neurodegenerative disorders like schizophrenia and Alzheimer’s disease [51,52,53,54,55,56,57,58]. It is thus particularly relevant to investigate possible OE cytoarchitectonic and biochemical alterations in patients inflicted with brain aging and mental disorders. Unlike the CNS, the OE is accessible for endoscopy and biopsy examinations, and OE mucus/swabs can be readily obtained and tested for cytological or biochemical abnormalities. 

## References

[1] Schultze M. (1856). Über die endigungsweise des geruchsnerven und die epithelialgebilde der nasenschleimhaut. Monatsberichte der Konigl Preußs. Akad der Wissen Berlin.

[2] Allison A.C. (1953). The morphology of the olfactory system in vertebrates. Biol. Rev..

[3] Breipohl W., Laugwitz H.J., Bornfeld N. (1974). Topological relations between the dendrites of olfactory sensory cells and sustentacular cells in different vertebrates. An ultrastructural study. J. Anat..

[4] Suzuki Y., Takeda M., Farbman A.I. (1996). Supporting cells as phagocytes in the olfactory epithelium after bulbectomy. J. Comp. Neurol..

[5] Schwob J.E. (2002). Neural regeneration and the peripheral olfactory system. Anat. Rec..

[6] Menco B.P.H.M., Morrison E.E., Doty R. (2003). Morphology of the mammalian olfactory epithelium: Form, fine structure and pathology. Handbook of Olfaction and Gustation.

[7] Asan E., Drenckhahn D. (2005). Immunocytochemical characterization of two types of microvillar cells in rodent olfactory epithelium. Histochem. Cell Biol..

[8] Elsaesser R., Paysan J. (2007). The sense of smell, its signalling pathways, and the dichotomy of cilia and microvilli in olfactory sensory cells. BMC Neurosci..

[9] Steinke A., Meier-Stiegen S., Drenckhahn D., Asan E. (2008). Molecular composition of tight and adherens junctions in the rat olfactory epithelium and fila. Histochem. Cell Biol..

[10] Hegg C.C., Irwin M., Lucero M.T. (2009). Calcium store-mediated signaling in sustentacular cells of the mouse olfactory epithelium. Glia.

[11] Liang F. (2018). Olfactory receptor neuronal dendrites become mostly intra-sustentacularly enwrapped upon maturity. J. Anat..

[12] Buck L.B. (2005). Unraveling the sense of smell (Nobel lecture). Angew. Chem. Int. Ed. Engl..

[13] Mori K., von Campenhause H., Yoshihara Y. (2000). Zonal organization of the mammalian main and accessory olfactory systems. Philos. Trans. R. Soc. Lond. B Biol. Sci..

[14] Okano M., Takagi S.F. (1974). Secretion and electrogenesis of the supporting cell in the olfactory epithelium. J. Physiol..

[15] Suzuki Y., Takeda M., Obara N., Suzuki N., Takeichi N. (2000). Olfactory epithelium consisting of supporting cells and horizontal basal cells in the posterior nasal cavity of mice. Cell Tissue Res..

[16] Hassenklöver T., Kurtanska S., Bartoszek I., Junek S., Schild D., Manzini I. (2008). Nucleotide-induced Ca^2+^ signaling in sustentacular supporting cells of the olfactory epithelium. Glia.

[17] Tang J., Tang J.H., Ling E.A., Wu Y., Liang F. (2009). Juxtanodin in the rat olfactory epithelium: Specific expression in sustentacular cells and preferential subcellular positioning at the apical junctional belt. Neuroscience.

[18] Graziadei P.P.C., Graziadei G.A.M. (1979). Neurogenesis and neuron regeneration in the olfactory system of mammals. I. Morphological aspects of differentiation and structural organization of the olfactory sensory neurons. J. Neurocytol..

[19] Shipley M.T., Ennis M., Puche A.C., Paxinos G. (2004). Olfactory system. The Rat Nervous System.

[20] Ashwell K., Watson C., Paxinos G., Puelles L. (2012). The olfactory system. The Mouse Nervous System.

[21] Brann J.H., Firestein S.J. (2014). A lifetime of neurogenesis in the olfactory system. Front. Neurosci..

[22] Kwon B.S., Kim M.K., Kim W.H., Pyo J.S., Cheon Y.H., Cha C.I., Nam S.Y., Baik T.K., Lee B.L. (2005). Age-related changes in microvillar cells of rat olfactory epithelium. Neurosci. Lett..

[23] Montani G., Tonelli S., Elsaesser R., Paysan J., Tirindelli R. (2006). Neuropeptide Y in the olfactory microvillar cells. Eur. J. Neurosci..

[24] Lemons K., Fu Z., Aoudé I., Ogura T., Sun J., Chang J., Mbonu K., Matsumoto I., Arakawa H., Lin W. (2017). Lack of TRPM5-expressing microvillous cells in mouse main olfactory epithelium leads to impaired odor-evoked responses and olfactory-guided behavior in a challenging chemical environment. eNeuro.

[25] Genovese F., Tizzano M. (2018). Microvillous cells in the olfactory epithelium express elements of the solitary chemosensory cell transduction signaling cascade. PLoS ONE.

[26] Graziadei P.P.C., Beidler L.M. (1971). The olfactory mucosa of vertebrates. Olfaction. Handbook of Sensory Physiology.

[27] Morrison E.E., Costanzo R.M. (1992). Morphology of olfactory epithlium in humans and other vertebrates. Microsc. Res. Tech..

[28] Nomura T., Takahashi S., Ushiki T. (2004). Cytoarchitecture of the normal rat olfactory epithelium: Light and scanning electron microscopic studies. Arch. Histol. Cytol..

[29] Ma M., Shepherd G.M. (2000). Functional mosaic organization of mouse olfactory receptor neurons. Proc. Natl. Acad. Sci. USA.

[30] Barral J.-P., Croibier A. (2009). Chapter 10: Olfactory nerve. Manual Therapy for the Cranial Nerves.

[31] Farbman A.I. (1992). Cell Biology of Olfaction.

[32] Smutzer G.S., Doty R.L., Arnold S.E., Trojanowski J.Q., Doty R. (2003). Olfactory system neuropathology in Alzheimer’s disease, Parkinson’s disease, and schizophrenia. Handbook of Olfaction and Gustation.

[33] Standring S. (2008). Gray’s Anatomy: The Anatomical Basis of Clinical Practice.

[34] Salazar I., Sanchez-Quinteiro P., Barrios A.W., López Amado M., Vega J.A. (2019). Anatomy of the olfactory mucosa. Handb. Clin. Neurol..

[35] Menco B.P.H.M. (1980). Qualitative and quantitative freeze-fracture studies on olfactory and nasal respiratory epithelial surfaces of frog, ox, rat, and dog III. Tight-junctions. Cell Tissue Res..

[36] Wolburg H., Wolburg-Buchholz K., Sam H., Horvát S., Deli M.A., Mack A.F. (2008). Epithelial and endothelial barriers in the olfactory region of the nasal cavity of the rat. Histochem. Cell Biol..

[37] Moran D.T., Rowley J.C., Jafek B.W., Lovell M.A. (1982). The fine structure of the olfactory mucosa in man. J. Neurocytol..

[38] Vogalis F., Hegg C.C., Lucero M.T. (2005). Electrical coupling in sustentacular cells of the mouse olfactory epithelium. J. Neurophysiol..

[39] Zhang C.B. (2010). Gap junctions in olfactory neurons modulate olfactory sensitivity. BMC Neurosci..

[40] Roskams A.J., Cai X., Ronnet G.V. (1998). Expression of neuron-specific beta-III tubulin during olfactory neurogenesis in the embryonic and adult rat. Neuroscience.

[41] Czesnik D., Schild D., Kuduz J., Manzini I. (2007). Cannabinoid action in the olfactory epithelium. Proc. Natl. Acad. Sci. USA.

[42] Breunig E., Manzini I., Piscitelli F., Gutermann B., Di Marzo V., Schild D., Czesnik D. (2010). The endocannabinoid 2-arachidonoyl-glycerol controls odor sensitivity in larvae of *Xenopus laevis*. J. Neurosci..

[43] Ogura T., Szebenyi S.A., Krosnowski K., Sathyanesan A., Jackson J., Lin W. (2011). Cholinergic microvillous cells in the mouse main olfactory epithelium and effect of acetylcholine on olfactory sensory neurons and supporting cells. J. Neurophysiol..

[44] Hutch C.R., Hegg C.C. (2016). Cannabinoid receptor signaling induces proliferation but not neurogenesis in the mouse olfactory epithelium. Neurogenesis.

[45] Fletcher R.B., Das D., Gadye L., Street K.N., Baudhuin A., Wagner A., Cole M.B., Flores Q., Choi Y.G., Yosef N. (2017). Deconstructing olfactory stem cell trajectories at single-cell resolution. Cell Stem Cell.

[46] Coleman J.H., Lin B., Louie J.D., Peterson J., Lane R.P., Schwob J.E. (2019). Spatial determination of neuronal diversification in the olfactory epithelium. J. Neurosci..

[47] Li H., Li T., Horns F., Li J., Xie Q., Xu C., Wu B., Kebschull J.M., McLaughlin C.N., Kolluru S.S. (2020). Single-cell transcriptomes reveal diverse regulatory strategies for olfactory receptor expression and axon targeting. Curr. Biol..

[48] Craig A.D. (2002). How do you feel? Interoception: The sense of the physiological condition of the body. Nat. Rev. Neurosci..

[49] Murinson B.B., Griffin J.W. (2004). C-fiber structure varies with location in peripheral nerve. J. Neuropathol. Exp. Neurol..

[50] Harty B.L., Monk K.R. (2017). Unwrapping the unappreciated: Recent progress in Remak Schwann cell biology. Curr. Opin. Neurobiol..

[51] Arnold S.E., Smutzer G.S., Trojanowski J.Q., Moberg P.J. (1998). Cellular and molecular neuropathology of the olfactory epithelium and central olfactory pathways in Alzheimer’s disease and schizophrenia. Ann. N. Y. Acad. Sci..

[52] Doty R.L. (2012). Olfaction in Parkinson’s disease and related disorders. Neurobiol. Dis..

[53] Buron E., Bulbena A. (2013). Olfaction in affective and anxiety disorders: A review of the literature. Psychopathology.

[54] Casjens S., Eckert A., Woitalla D., Ellrichmann G., Turewicz M., Stephan C., Eisenacher M., May C., Meyer H.E., Brüning T. (2013). Diagnostic value of the impairment of olfaction in Parkinson’s disease. PLoS ONE.

[55] Seligman S.C., Kamath V., Giovannetti T., Arnold S.E., Moberg P.J. (2013). Olfaction and apathy in Alzheimer’s disease, mild cognitive impairment, and healthy older adults. Aging Ment. Health.

[56] Auster T.L., Cohen A.S., Callaway D.A., Brown L.A. (2014). Objective and subjective olfaction across the schizophrenia spectrum. Psychiatry.

[57] Doty R.L., Hawkes C.H., Good K.P., Duda J.E., Doty R.L. (2015). Odor perception and neuropathology in neurodegenerative diseases and schizophrenia. Handbook of Olfaction and Gustation.

[58] Field T. (2015). Smell and taste dysfunction as early markers for neurodegenerative and neuropsychiatric diseases. J. Alzheimers Dis. Parkinsonism.

[59] Holbrook E.H., Wu E., Curry W.T., Lin D.T., Schwob J.E. (2011). Immunohistochemical characterization of human olfactory tissue. Laryngoscope.

[60] Melendez-Vasquez C.V., Rios J.C., Zanazzi G., Lambert S., Bretscher A., Salzer J.L. (2001). Nodes of Ranvier form in association with ezrin-radixin-moesin (ERM)-positive Schwann cell processes. Proc. Natl. Acad. Sci. USA.

[61] Gatto C.L., Walker B.J., Lambert S. (2003). Local ERM activation and dynamic growth cones at Schwann cell tips implicated in efficient formation of nodes of Ranvier. J. Cell Biol..

[62] Nawaz S., Sánchez P., Schmitt S., Snaidero N., Mitkovski M., Velte C., Brückner B.R., Alexopoulos I., Czopka T., Jung S.Y. (2015). Actin filament turnover drives leading edge growth during myelin sheath formation in the central nervous system. Dev. Cell.

[63] Samanta J., Salzer J.L. (2015). Myelination: Actin disassembly leads the way. Dev. Cell.

[64] Zuchero J.B., Fu M.M., Sloan S.A., Ibrahim A., Olson A., Zaremba A., Dugas J.C., Wienbar S., Caprariello A.V., Kantor C. (2015). CNS myelin wrapping is driven by actin disassembly. Dev. Cell.

[65] Maurya D.K., Henriques T., Marini M., Pedemonte N., Galietta L.J., Rock J.R., Harfe B.D., Menini A. (2015). development of the olfactory epithelium and nasal glands in TMEM16A^-/-^ and TMEM16A^+/+^ mice. PLoS ONE.

[66] Zhang B., Cao Q., Guo A., Chu H., Chan Y.G., Buschdorf J.P., Low B.C., Ling E.A., Liang F. (2005). Juxtanodin: An oligodendroglial protein that promotes cellular arborization and 2’,3’-cyclic nucleotide-3’-phosphodiesterase trafficking. Proc. Natl. Acad. Sci. USA.

[67] Brockschnieder D., Sabanay H., Riethmacher D., Peles E. (2006). Ermin, a myelinating oligodendrocyte-specific protein that regulates cell morphology. J. Neurosci..

[68] Meng J., Xia W., Tang J.H., Tang B.L., Liang F. (2010). Dephosphorylation-dependent inhibitory activity of juxtanodin on filamentous actin disassembly. J. Biol. Chem..

[69] Ruskamo S., Chukhlieb M., Vahokoski J., Bhargav S.P., Liang F., Kursula I., Kursula P. (2012). Juxtanodin is an intrinsically disordered F-actin-binding protein. Sci. Rep..

[70] Liang F., Hwang J.H., Tang N.W., Hunziker W. (2018). Juxtanodin in retinal pigment epithelial cells: Expression and biological activities in regulating cell morphology and actin cytoskeleton organization. J. Comp. Neurol..

[71] Matsumoto B., Defoe D.M., Besharse J.C. (1987). Membrane turnover in rod photoreceptors: Ensheathment and phagocytosis of outer segment distal tips by pseudopodia of the retinal pigment epithelium. Proc. R. Soc. Lond. B Biol. Sci..

[72] Buck L.B., Axel R. (1991). A novel multigene family may encode odorant receptors: A molecular basis for odor recognition. Cell.

[73] Fleischer J., Breer H., Strotmann J. (2009). Mammalian olfactory receptors. Front. Cell. Neurosci..

[74] Johnson M.A., Tsai L., Roy D.S., Valenzuela D.H., Mosley C., Magklara A., Lomvardas S., Liberles S.D., Barnea G. (2012). Neurons expressing trace amine-associated receptors project to discrete glomeruli and constitute an olfactory subsystem. Proc. Natl. Acad. Sci. USA.

[75] Ihara S., Yoshikawa K., Touhara K. (2013). Chemosensory signals and their receptors in the olfactory neural system. Neuroscience.

[76] Bear D.M., Lassance J.M., Hoekstra H.E., Datta S.R. (2016). The evolving neural and genetic architecture of vertebrate olfaction. Curr. Biol..

